# Intussusceptions as acute abdomen caused by Burkitt lymphoma: a case report

**DOI:** 10.1186/1757-1626-2-9322

**Published:** 2009-12-14

**Authors:** Faton T Hoxha, Shemsedin I Hashani, Avdyl S Krasniqi, Fisnik I Kurshumliu, Driton S Komoni, Shpresa M Hasimja, Mehmet Maxhuni

**Affiliations:** 1 University Clinical Centre of Kosova, Rrethi Spitalit str p n, Prishtina 10000, Republic of Kosovo; 2 Medical Faculty, University of Prishtina, Mother Theresa str pn, Prishtina 10000, Republic of Kosovo

## Abstract

**Introduction:**

Burkitt's lymphoma is a highly malignant, aggressive and rapidly growing B-cell neoplasm, which has low long-term survival rates. The abdomen is the most frequent onset site of non endemic Burkitt's lymphoma. Symptoms are often misleading and make diagnosis difficult. Ileum intussusception as acute abdomen caused by Burkitt lymphoma is rare.

**Case presentation:**

We are presenting a case of a 16 year-old male with acute abdomen, which three weeks prior initially has been surgically treated for acute appendicitis and Meckel diverticulitis. Following this was a second urgent operation of ileoileal intusussception caused by Burkitt lymphoma. Right extended haemicolectomy was performed.

**Conclusion:**

Affected terminal ileum by Burkitt's lymphoma may mimic clinically acute appendicitis and investigation tools sometimes may not provide proper diagnosis.

Complete resection results in improved survival.

## Introduction

First described by Dennis Burkitt in 1958, Burkitt lymphoma (BL) is a highly aggressive non-Hodgkin lymphoma (NHL) often presenting in extra nodal sites or as an acute leukemia [1].

Three variants have been described: endemic (largely found in Africa), sporadic (non-endemic) subsequently described outside Africa, affecting mainly abdominal viscera and third variant, immunodeficient patients. Burkitt's lymphoma is usually diagnosed in children and young adults, and to a lesser extent in middle aged adults. BL is a B-cell lymphoma genetically characterized by a chromosomal translocation that results in deregulation of the c-MYC oncogene [2-4].

In endemic areas, usually involves the facial bones, particularly the jaw, maxilla, and orbit, especially in young children, associated with Epstein-Barr virus (EBV) infection, as well as frequent concomitant malaria infection [5,6].

In comparison, the sporadic form tends to present in the lymphoid tissues of the gut, often presenting as masses in the Waldeyer ring or the terminal ileum, or even with involvement of abdominal organs with the most involvement of the distal ileum, caecum or mesentery. Bone marrow involvement is commonly seen in progressive disease [6-8].

## Case presentation

We are presenting a case with acute abdomen, i.e. ileoileal intusussception, caused by Burkitt lymphoma. A sixteen-year-old Caucasian Kosovar boy, presented in the surgical emergency clinic as acute abdomen, with abdominal pain, nausea, vomiting, and problems with defecation and flatulence. The symptoms started three days ago, worsening on admission day.

The patient's height was 178 cm, and his weight was 70 kg. His vital signs were: blood pressure 120/80 mmHg; pulse rate: 76 beats/min; respiratory rate 20/min; body temperature 37.1°C.

During physical examination, a distended, diffusely tender and painful abdomen with lower abdomen rebound was revealed. The patient's skin was pale with sweats.

**Figure 1:**
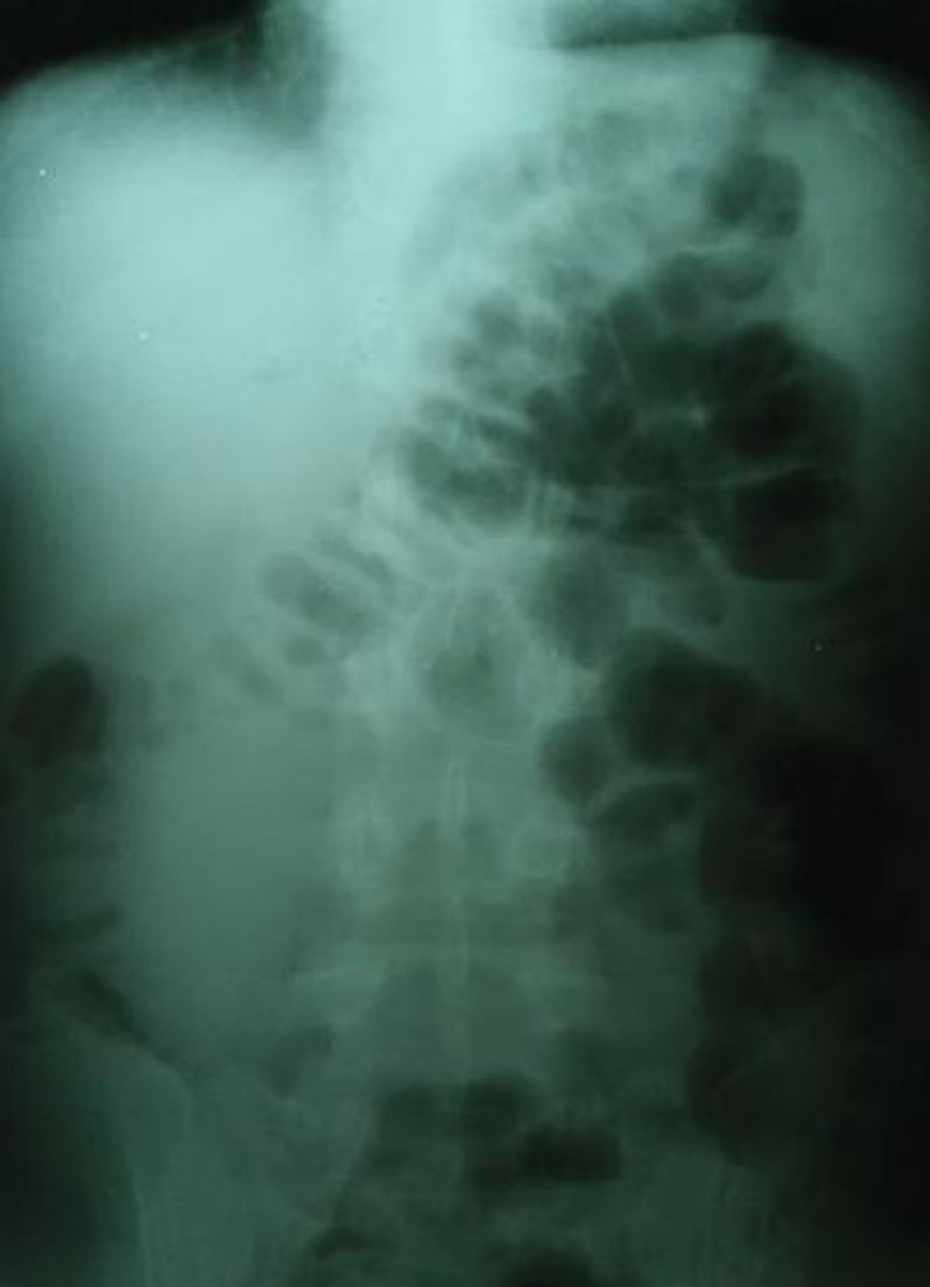
**Abdominal native radiography**.

Plane abdominal radiography showed mechanical obstruction. (Fig. 1)

The emergency laboratory tests presented as follows: Red blood cells (RBC): 4.61 × 10^9^/L; white blood cells (WBC): 7.3 × 10^9^/L; Hemoglobin (Hgb): 128 g/L; Hematocrit (Htc): 0.42; blood sugar: 5.42 mmol/L; blood urea nitrogen (BUN): 2.6 mmol/L; serum creatinine: 88 μmol/L; BUN/Creat ratio: 8.5; total protein: 72 g/L; albumin: 36 g/l; ALP: 60 IU/L; ALT: 26 IU/L; AST:40 IU/L; direct bilirubin: 5.8 μmol/L; total bilirubin: 20.5 μmol/L; indirect bilirubin: 14.7 μmol/L; Gama GT: 26 IU/L; C-reactive protein: 15.6 mg/L; serum amylase: 30 U/L: electrolytes: Na: 138 mmol/L; K: 3.75 mmol/L; Cl: 102 mmol/L; urinalysis: 2-4 Leucocytes, some bacteria and some uric acid crystals. Blood group: O Rh(D) poz.

Three weeks prior he had been surgically treated as acute abdomen caused by acute appendicitis and Mckelly Diverticulitis. Appendectomy and short resection of the ileum with diverticulum, and end-to-end anastomosis was performed. The immediate post operative period went well. After his discharge, his second hospital admission was two weeks after operation with abdominal pain and constipation problems which released spontaneously after two days.

At his third admission as acute abdomen, urgent laparotomy was performed in general endo tracheal anesthesia on the day after admission. Intra operative findings revealed small bowel dilatation, with intussusceptions of the terminal ileum, 2 cm from ileocecac valve. There were multiple enlarged mesenteric lymph nodes at the meso of the terminal ileum, ascending and the transverse colon, without palpable liver metastases.

**Figure 2:**
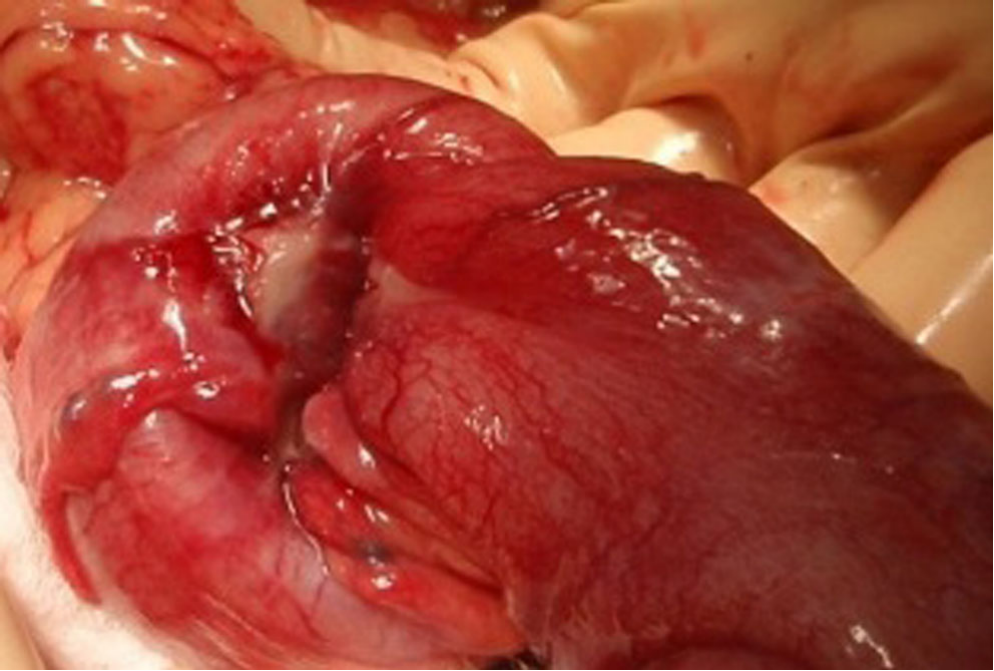
**Terminal ileum intussusception caused by Burkitt lymphoma**.

**Figure 3:**
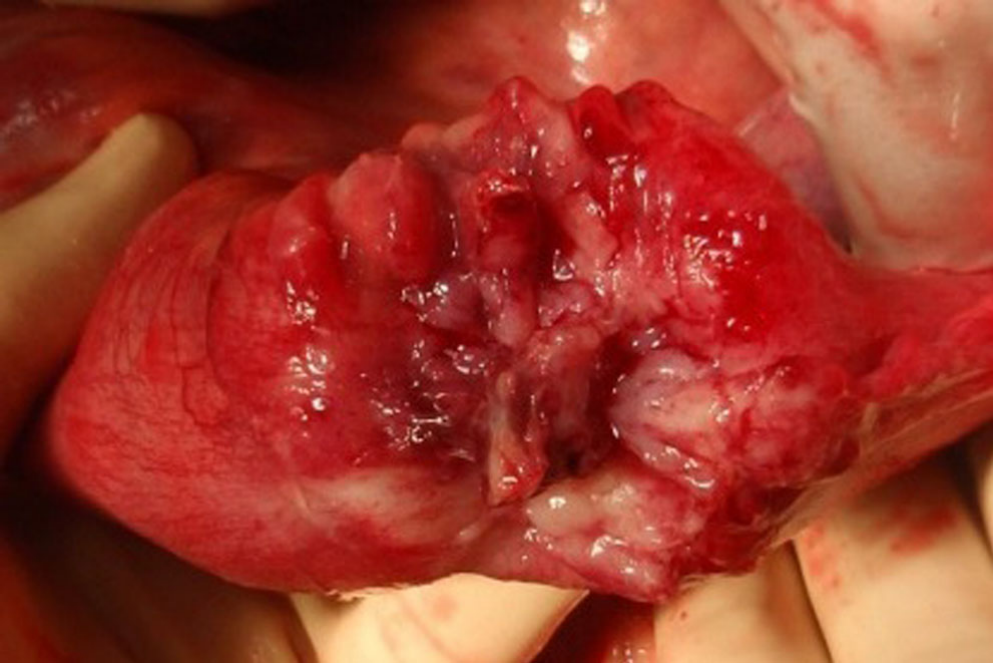
**Des intussusception of terminal ileum with Burkitt Lymphoma**.

**Figure 4:**
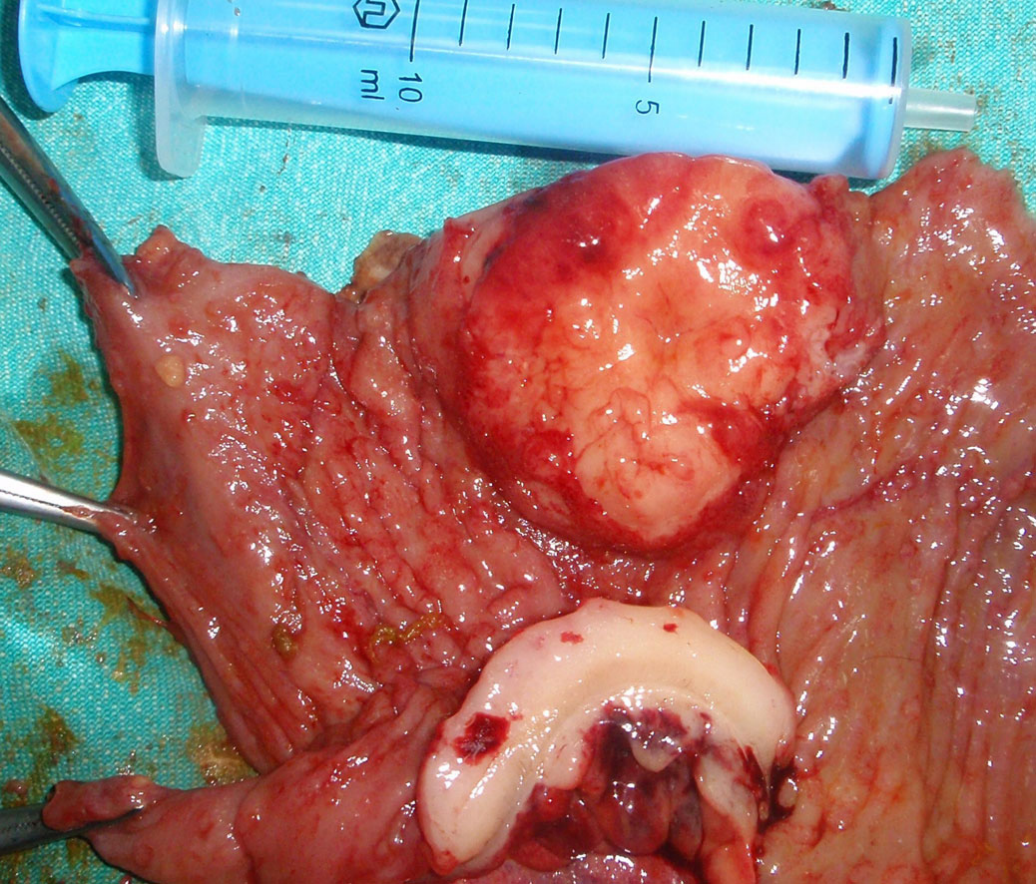
**Macroscopic view of opened terminal ileum and Burkitt lymphoma**.

We have done des intussusceptions caused by tumor from the wall of the ileum. (Fig. 2, 3, 4)

Adhesions at the anastomosis, and 60 cm above that, were released. The right extended haemicolectomy with end to end ileum-transverse anastomosis was performed. Post operative period went well; blood pressure was 120/80 mmHg, pulse rate beats: 65/min; temp.: 36.7°C. The wound healed per primam. He was treated with frozen fresh plasma (several doses-seven), red blood cells (one dose); antibiotic, analgesics, H2 blockers, vitamins, amino acids, human albumins. He was discharged on the 10^th ^postoperative days, with good bowel movements. He was oriented to the Hematology Department for further treatment, for Burkitt Lymphoma with chemotherapy.

**Figure 5:**
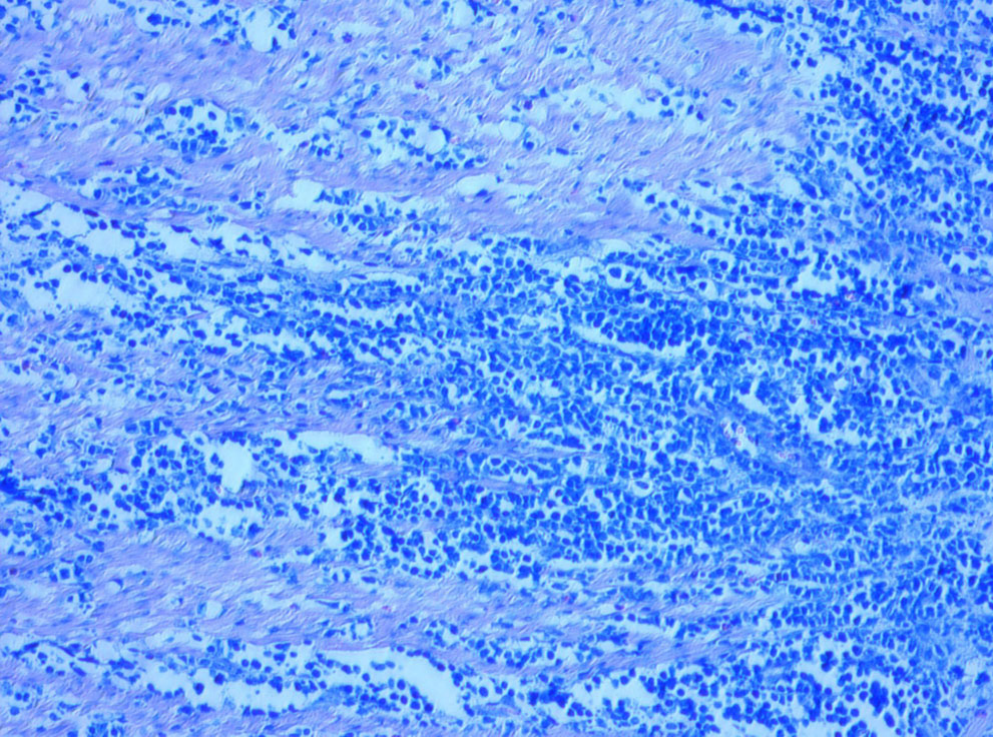
**Solid sheets of medium sized neoplastic lymphocytes infiltrating between layers of muscularis propria (×10; Giemsa stain)**.

**Figure 6:**
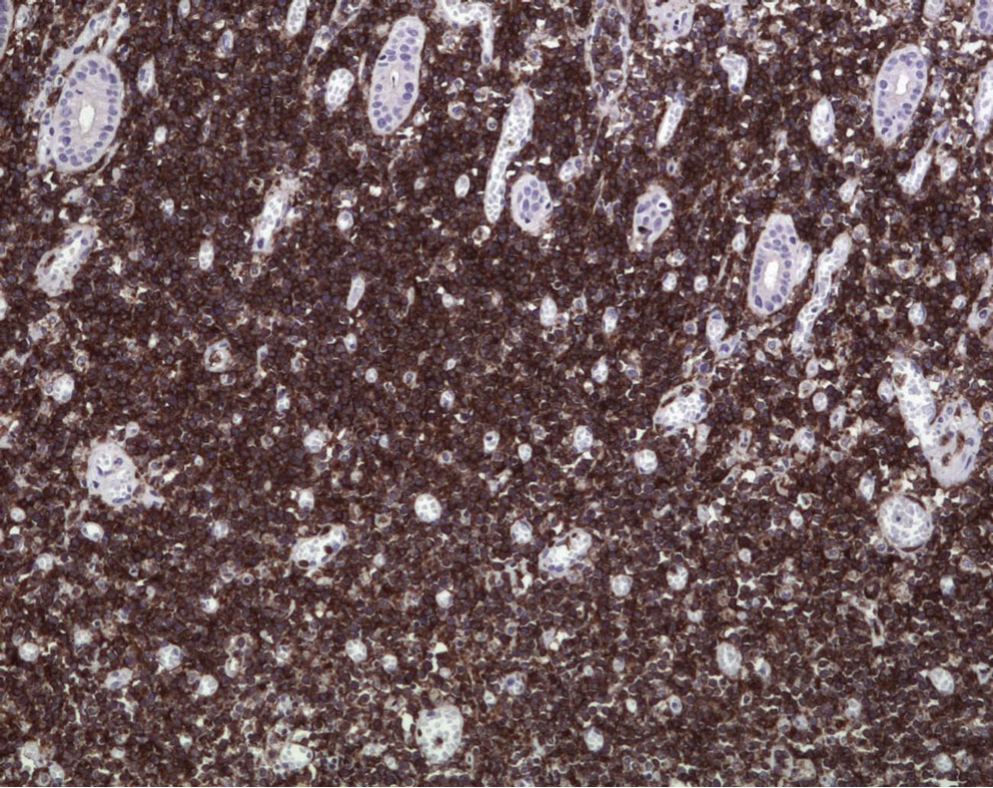
**Pronounced membranous immunoreactivity for CD10 of the neoplastic B-cells (×20; Immunoperoxidase)**.

**Figure 7:**
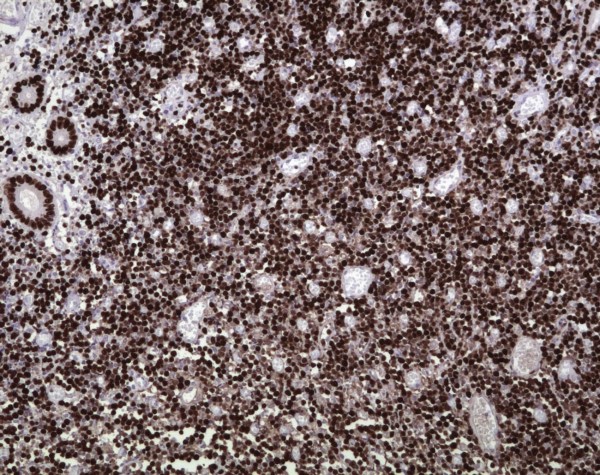
**A proliferation index of over 90% of neoplastic cells as determined by MIB-1 immunohistochemistry (×10; Immunoperoxidase)**.

**Figure 8:**
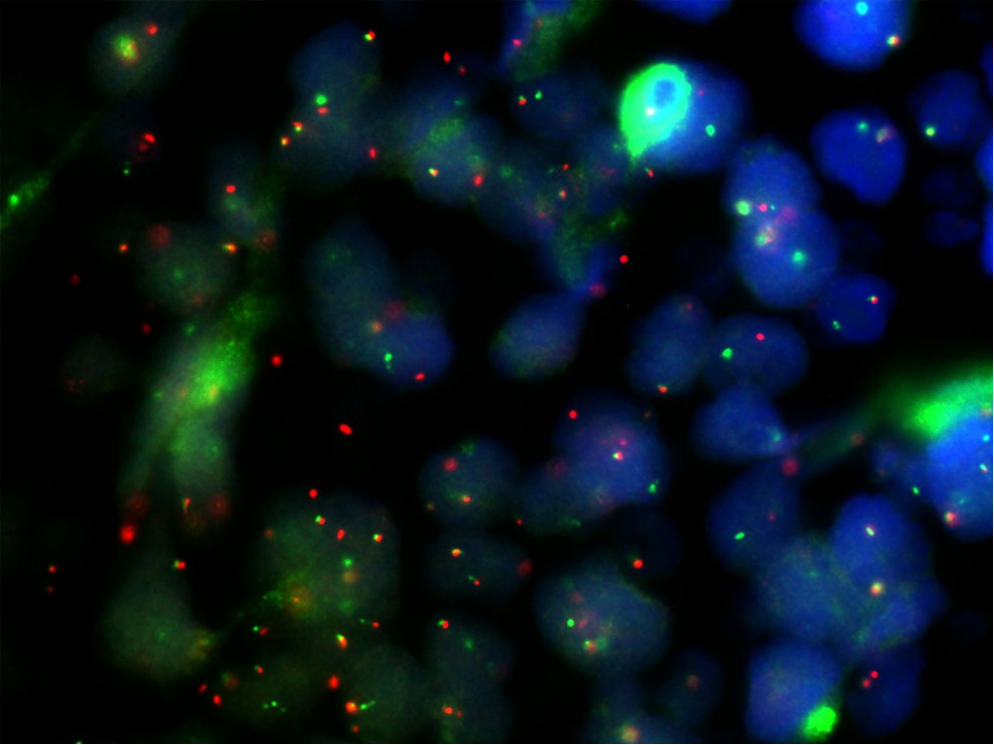
**c-myc translocation as determined by FISH analysis (fusion)**.

**Figure 9:**
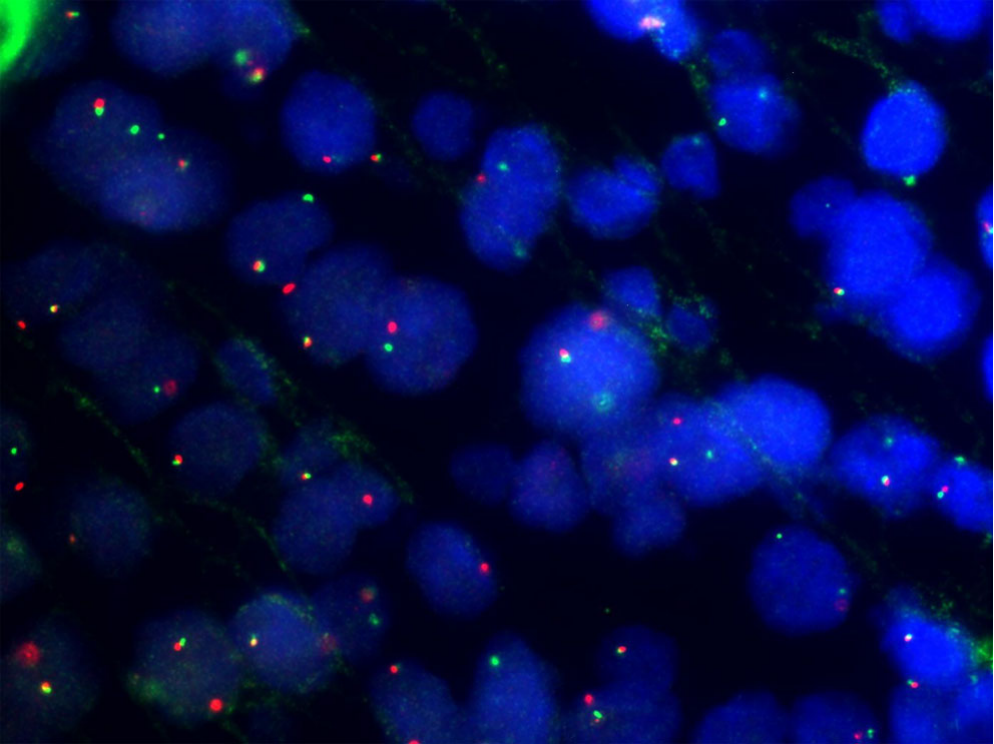
**c-myc translocation as determined by FISH analysis (split)**.

HP opinion: Giemsa staining demonstrated neoplastic lymphocytes infiltration. Immunochemical testing was positive for Burkitt lymphoma (CD10, CD20, CD34, Bcl-2, Ki67, IgM, MIB-1 with a proliferation index of over 90% of neoplastic cells) and c-myc translocation determined by FISH analysis (fusion and split). (Fig. 5, 6, 7, 8, 9)

## Discussion

Sporadic Burkitt's lymphoma accounts for 1%-2% of lymphoma in adults and up to 40% of lymphoma in children in the United States and Western Europe [2].

Estimated incidence ranges from one case per million for children less than ten years old, 0.7 per million from 10 to 20 and 0.6 per million from 20 to 30, and rare in patients older than 30 [9,10].

Burkitt's lymphoma is an aggressive, highly malignant and rapidly growing B-cell neoplasm frequently presenting onset in the abdomen in non-endemic Burkitt's lymphoma regions. Intussusception caused by Burkitt lymphoma, as a cause of acute abdomen, is rare, with symptoms which often mislead and make diagnosis more difficult [11].

The rapidity of volumetric doubling of this neoplasm frequently justifies an acute abdomen presentation that may mime other less-rare diseases [6,11].

The clinical presentation on undiagnosed Burkitt lymphoma is very non-specific, which makes this a difficult condition to diagnose.

The sporadic form commonly presents with abdominal swelling as a large mesenteric, retroperitoneal or pelvic mass, tenderness, pain or fullness. Some patients present with symptoms of bowel obstruction secondary to ileo-caecal intussusception caused by tumor growth, obstruction or bleeding, mimicking acute appendicitis [6,10].

Our patient had a clinical presentation of acute abdomen, with abdominal pain, distended abdomen, vomiting, and no flatulence with intestinal obstruction.

Burkitt's lymphoma is more common in males than females [10,12-15].

In reviews that includes significant numbers of adult patients with abdominal Burkitt's lymphoma, about 33% to 50% of the patients were older than 20 [10,15].

Magrath's review included 97% patients younger than 15 [16].

Burkitt's lymphoma occurring in adults, involving the terminal ileum with ileal or ileocolic intussusception's has been reported as acute abdomen required an emergency surgery reported as case or as small numbers in series, treated with small bowel resection or right haemicolectomy [10,11,14,15,17-19].

Our case presentation is the first case of Burkitt's lymphoma with abdominal involvement, causing ileo-ileal intussusception, diagnosed and treated with extensive surgical resection here in Kosova.

Surgery is required in the treatment of Burkitt's lymphoma to confirm the diagnosis and to relieve the common presenting symptoms of intestinal obstruction, abdominal mass, intussusception, or acute abdomen. Complete resection is associated with improved survival.

Some reports demonstrated higher survival rate (58%-89%) in patients having extensive surgical resection versus for patients having only partial or incomplete resection (40%-45%) at 2-5 years [[10,15], and [16]].

However, this may merely reflect the fact that less disease is more easily completely resected [20].

These authors broadened the role of surgery by suggesting that, even in patients with disseminated disease, the surgeon plays an important role in the secondary management of the disease.

## Conclusion

Affected terminal ileum by Burkitt's lymphoma may mimic clinically acute appendicitis and investigative tools sometimes may not provide proper diagnosis.

Complete resection results in an improved patient survival.

## Patient's perspective

Symptoms like abdominal pain, nausea, vomiting, and problems with defecation and flatulence they started three days ago, worsening on admission day in Hospital. My skin was pale and sweats. During physical examination, my belly was distended, diffusely tender and painful. Plane abdominal radiography showed mechanical obstruction.

Three weeks prior I had been surgically treated as acute abdomen caused by acute appendicitis and Mckelly Diverticulitis. Appendectomy and short resection of the ileum with diverticulum, and end-to-end anastomosis was performed. The immediate post operative period went well. After discharge, my second hospital admission was two weeks after operation with abdominal pain and constipation problems which released spontaneously after two days.

At my third admission as acute abdomen, urgent laparotomy was performed in general endo tracheal anesthesia on the day after admission.

The right extended haemicolectomy with end to end ileum-transverse anastomosis was performed. Post operative period went well; the wound healed per primam. I was discharged on the 10^th ^postoperative days, with good bowel movements, and oriented to the Hematology Department for further treatment, for Burkitt Lymphoma with chemotherapy.

Burkitt's lymphoma as sporadic form, and very rare in our country affecting abdomen in children and young adults, with extensive surgery and chemotherapy gives a good hope for the future of this patients.

## Consent

Written informed consent was obtained from the patient for publication of this case report and accompanying images. A copy of the written consent is available for review by the Editor-in-Chief of this journal.

## Competing interests

The authors declare that they have no competing interests.

## Authors' contributions

FTH, SHIH, DSK and SHIH performed the surgery and the general anesthesia.

FIK has made Histopathology. FTH designed the work, the other authors contributed equally to this work.
